# Evaluation of two different etorphine doses combined with azaperone in blesbok (*Damaliscus pygargus phillipsi*) immobilisation

**DOI:** 10.4102/jsava.v92i0.2161

**Published:** 2021-08-24

**Authors:** Eugenio Gaudio, Liesel L. Laubscher, Leith C.R. Meyer, Louwrens C. Hoffman, Jacobus P. Raath, Silke Pfitzer

**Affiliations:** 1 Department of Animal Medicine Production and Health, School of Agricultural Sciences and Veterinary Medicine, University of Padova, Padova, Italy; 2 Department of Animal Sciences, Faculty of Agrisciences, Stellenbosch University, Stellenbosch, South Africa; 3 Department of Research and Development, Wildlife Pharmaceuticals (Pty) Ltd., White River, South Africa; 4 Centre for Veterinary Wildlife Studies, Faculty of Veterinary Sciences, University of Pretoria, Ondestepoort, South Africa; 5 Department of Paraclinical Sciences, Faculty of Veterinary Science, University of Pretoria, Onderstepoort, South Africa; 6 Centre for Nutrition and Food Science, Queensland Alliance for Agriculture and Food Innovation, University of Queensland, Brisbane, Australia; 7 Wildlife Pharmaceuticals, White River, South Africa; 8 School of Biology and Environmental Sciences, University of Mpumalanga, Nelspruit, South Africa

**Keywords:** anaesthesia, antelope, azaperone, chemical immobilisation, etorphine, wildlife

## Abstract

Chemical immobilisation is essential for veterinarians to perform medical procedures in wild African ungulates. Potent opioids combined with neuroleptic drugs are most often used for this purpose. The present study aimed at comparing the quality of immobilisation and effects on physiological variables between a high (high etorphine-azaperone [HE]: 0.09 mg kg^–1^) and low etorphine dose (low etorphine-azaperone [LE]: 0.05 mg kg^–1^), both combined with azaperone (0.35 mg kg^–1^), in 12 adult female boma-acclimatised blesbok. It was hypothesised that a reduction in etorphine’s dose in combination with azaperone would result in less cardiorespiratory impairment but likely worsen the quality of immobilisation. Both treatments resulted in rapid induction and recovery times. Overall inter-treatment differences occurred in pulse rate (HE and LE: 52 ± 15 and 44 ± 11 beats minute^–1^, *p* < 0.0001), respiratory rate (HE and LE: 15 ± 4 and 17 ± 4 breaths minute^–1^, *p* < 0.006), partial pressure of exhaled carbon dioxide (HE and LE: 62.0 ± 5.0 and 60.0 ± 5.6 millimetre of mercury [mmHg], *p* < 0.028) and arterial carbon dioxide (HE and LE: 58.0 ± 4.5 and 55.0 ± 3.9 mmHg, *p* < 0.002). Both HE and LE led to bradycardia, hypertension and marked hypoxia to a similar extent. Furthermore, quality of induction, immobilisation and recovery were similar in both treatments. The role of azaperone in the development of cardiorespiratory compromise and gas exchange impairment that occurred when these combinations were used is still unclear. Further studies are recommended to elucidate drug- and dose-specific physiological effects in immobilised antelope.

## Introduction

Blesbok (*Damaliscus pygargus phillipsi*) are gregarious, medium-sized antelope that inhabit the open grassland of southern Africa and are a favoured species amongst wildlife ranchers, resulting in regular sale and translocations of live animals (Frost 2015).

Chemical immobilisation is essential to perform medical procedures and for the capture and translocation of wildlife that would otherwise be unapproachable and impossible to handle. Potent opioids are most commonly used for immobilisation because of their rapid onset and complete reversibility (Kock & Burroughs 2012). The most extensively used potent opioid is etorphine, a µ-, κ- and δ-opioid receptor agonist (Burroughs 1993; Gaudio et al. 2020a; Gutstein & Akil 2006; Kock & Burroughs 2012; Williams & Riedesel 1987). Unfortunately, several side effects are common with the use of potent opioids, including cardiovascular and respiratory compromise, tachycardia, hypertension and poor muscle relaxation (Grimm et al. 2015; Kock & Burroughs 2012).

With the aim of improving the quality of immobilisation and reducing the dose of potent opioids, benzodiazepines, α_2_-agonists or butyrophenone derivatives, such as azaperone, are often combined in a dart with these opioids (Kock & Burroughs 2012). Azaperone is a neuroleptic drug, which binds to dopamine (D_2_) receptors, exerting an antagonistic activity, which leads to tranquilisation and potentiation of opioid-based immobilisation (Kock & Burroughs 2012; Riviere & Papich 2009; Swan 1993). It has been shown to be fully effective within 15–20 min following intramuscular (IM) injection, and in horses its effect lasts up to 6 h (Lees & Serrano 1976). Butyrophenones are also believed to be beneficial in wildlife immobilisation because of their mild antagonistic activity on α_1_-adrenergic receptors, leading to peripheral vasodilation that can be used to counter hypertension (Riviere & Papich 2009).

Despite their extensive use, very few immobilising drug combinations have been scientifically assessed for quality of immobilisation and physiological effects in different antelope species; in particular, very little work has been carried out on blesbok (Du Plessis 2018; Semjonov et al. 2018; Pfitzer et al. 2019, 2021; Zeiler & Meyer 2017). Recently, the authors of this article compared the quality of immobilisation and cardiorespiratory effects of a high dose of etorphine alone (0.09 mg kg^-1^) and an etorphine (0.09 mg kg^-1^) azaperone (0.35 mg kg^-1^) combination in blesbok (Gaudio et al. 2020b). Results from this study revealed that both protocols cause significant cardiorespiratory compromise in the animals, which was worse with the etorphine–azaperone combination. However, the etorphine–azaperone drug combination provided a deeper plane of immobilisation, allowing for easier handling of the animals.

The present study aimed to investigate and compare two etorphine–azaperone drug combinations comprised of two different doses of etorphine (0.09 mg kg^-1^and 0.05 mg kg^-1^) and the same dose of azaperone (0.35 mg kg^-1^). The aim was to evaluate possible differences in effects on cardiorespiratory function and quality of immobilisation between the two combinations. It was hypothesised that the combination with the lower dose of etorphine would lead not only to less cardiorespiratory impairment but also a poorer quality of immobilisation than the higher etorphine dose-based combination.

## Materials and methods

### Animals and housing

The study was carried out at the Wildlife Pharmaceuticals Wildlife Research Facility. Twelve wild female blesbok were acclimatised at the research facility for 2 weeks before the start of the study. After 1 week of acclimatisation, the animals were darted for a preliminary assessment, which included physical examination, blood tests, weighing and ear tagging. The blesbok were kept in groups of 4 in 3 adjacent enclosures, where they were provided feed and water *ad libitum*.

### Drug combinations and study design

This research was performed as a blinded randomised crossover study. Each animal received each drug combination once with a 1-week washout period between treatments. A simple randomisation method was used (SAS version 9.3, SAS Institute, United States).

The treatments consisted of a high etorphine dose (0.09 mg kg^-1^) (Captivon 98˚, Wildlife Pharmaceuticals [Pty] Ltd., White River, South Africa [SA]) combined with azaperone (0.35 mg kg^-1^) (high etorphine-azaperone [HE]) (100 mg mL^-1^, Wildlife Pharmaceuticals [Pty] Ltd., White River, SA) and a lower etorphine dose (0.05 mg kg^-1^) combined with azaperone (0.35 mg kg^-1^) (low etorphine-azaperone [LE]). Naltrexone (Trexonil^®^, Wildlife Pharmaceuticals [Pty] Ltd., White River, SA) at a dose of 20 mg naltrexone per 1 mg etorphine was intravenously administered to antagonise the effects of etorphine at the end of each immobilisation.

### Immobilisation and monitoring

The animals were deprived of water and feed for 12 h before immobilisation. Immobilising drugs were administered with a 1-mL dart-syringe with a 1.91-cm barbed needle (Type ‘P’ RDD Device, Pneu-Dart. Inc., Williamsport, United States) projected from a gas-powered dart gun (X-Caliber, Pneu-Dart. Inc., Williamsport, United States). Time from darting to when the animal showed first signs of altered consciousness (time to first sign) and time from darting to when the animal was recumbent (induction time) were noted. A subjective score (induction score, refer to Table 1) was allocated to each animal for the induction phase, which was defined as the period from drug injection to recumbency. Times and scores were adapted from Gaudio et al. (2020b).

**TABLE 1:** Description of the scoring system used to categorise the quality of induction, immobilisation and recovery of blesbok immobilised with two etorphine–azaperone combinations.†

Score	1	2	3	4	5
Induction	Slight ataxia followed by animal taking one or two attempts to sit and/or lie in sternal recumbency without signs of excitement or falling over during the process. A smooth transition into lateral recumbency may follow shortly after (excellent)	Moderate ataxia followed by animal taking one or two attempts to sit and/or lie in sternal recumbency. The animal may stumble during the process (good)	Severe ataxia followed by animal making numerous attempts to sit or lie down. Animal stumbles and falls on numerous occasions before becoming recumbent. Reaction to external stimuli (fair)	Severe ataxia but the animal does not become recumbent and/or the animal stumbles and falls repeatedly. Animal not approachable, requires a second dose of drugs (poor)	-
Immobilisation	Re-dosing is required to achieve recumbency. Risk of injury to the handler (limited effect)	Spontaneous motor activity, struggling during manipulation, presence of anal and palpebral reflexes, responsive to painful stimuli, might vocalise, presence of nystagmus, chewing, ear movements and strong panniculus reflex (deep sedation)	Muscle rigidity, slow palpebral reflex, voluntary tail movements, central eye position. Might vocalise, some chewing, ear movements might be absent, weak nystagmus, attenuated panniculus and anal reflex. Animal can be handled safely (light immobilisation plane)	Smooth, complete relaxation, extractable tongue, loss of palpebral reflex and jaw tone, no involuntary tail movements, ventromedial eye position, no nystagmus, no panniculus and anal reflex, no reaction to blood sampling, safe handling (deep immobilisation plane)	Too deep, absent reflexes, cardiorespiratory depression (excessively deep)
Recovery	Stands in one or two attempts and is sufficiently recovered to walk with only slight ataxia. Recovery to walking occurs within 2 min following the administration of drug antagonist (excellent)	Some imbalance in sternal recumbency and requires more than two attempts to stand. Walks with moderate ataxia and lack of coordination. Recovery to walking occurs within 5 min following the administration of drug antagonist (good)	Animal remains in lateral recumbency for more than 5 min following the administration of drug antagonist, is not responsive to stimuli and makes no attempt to transition to sternal recumbency. Or, animal has a stormy recovery with marked ataxia with the potential for injury. May require sedation (poor)	Animal does not recover and eventually dies, or its conditions are such that it needs to be euthanised (unacceptable)	-

*Source*: Adapted from see the full reference list of the article Gaudio, E., Laubscher, L.L., Pfitzer, S., Raath, J.P., Hoffman, L.C. & Benedictis, G.M., 2020b, ‘Immobilisation quality and cardiopulmonary effects of etorphine alone compared with etorphine–azaperone in blesbok (Damaliscus pygargus phillipsi)’, *Veterinary Anaesthesia and Analgesia* 47(4), 528–536. https://doi.org/10.1016/j.vaa.2019.10.012, for more information.

HE, high etorphine-azaperone; LE, low etorphine-azaperone.

† , HE: 0.09 mg kg^-1^ etorphine and 0.35 mg kg^-1^ azaperone; LE: 0.05 mg kg^-1^ etorphine and 0.35 mg kg^-1^ azaperone.

After recumbency, the immobilised blesbok was moved onto a stretcher from the enclosure to a nearby shaded area. The animal was positioned in sternal recumbency on a table and its horns were held by a handler so that the head was elevated above the thorax and the neck aligned with the vertebral column. The blesbok was then blindfolded and earplugs were inserted into the external ear canal. Assessment of respiratory variables was performed by inserting an endotracheal tube (inner diameter 7 mm) into one nostril, secured at the level of the medial canthus of the eye by cuff inflation. A catheter was placed into the caudal auricular artery for assessment of blood pressure variables. When it was not possible to cannulate this artery, the median artery of the metacarpus was used as an alternative site. Evaluation of physiological variables and a subjective assessment of the quality of immobilisation (scoring system from Gaudio et al. 2020b) were performed at 5-min intervals, starting 5 min after recumbency and lasting for 40 min.

Respiratory rate (ƒ_R_) and end-tidal carbon dioxide (P_E_’CO_2_) (mainstream method; Capnostat, Respironics, Inc., Wallingford, United States) were measured by using a multi-parameter monitor (Cardell 9500 HD Veterinary Monitor, Midmark Corporation, United States [US]). Pulse rate (PR) and invasive blood pressure (mean, systolic and diastolic blood pressure: MAP, SAP, DAP, respectively) were measured by means of a portable monitor (IntraTorr, IntraVitals, United Kingdom) connected to a pre-calibrated Deltran II pressure transducer (Utah Medical, US). A pulse oximeter (Nonin PalmSat 2500, the Netherlands) with a reflectance probe attached to the skin under the tail was used to assess peripheral oxyhaemoglobin saturation (SpO_2_). Rectal temperature was measured by means of a thermometer (Hanna Checktemp 1, Hanna Instruments [Pty] Ltd., SA). Manual counts of breaths and heart auscultation were also performed to further confirm the accuracy of the electronic monitors. Arterial blood samples were anaerobically collected at 5, 10, 15, 20 and 30 min post-recumbency from the arterial catheters into heparinised syringes. Blood gas analysis was performed within 5 min from collection on these samples by using a portable analyser (EPOC Reader Blood Analysis and pre-calibrated EPOC BGEM smart cards, Epocal, Kyron Laboratories, SA). Variables assessed were: arterial partial pressure of oxygen (PaO_2_), arterial partial pressure of carbon dioxide (PaCO_2_), arterial blood pH, bicarbonate (HCO_3_^–^), base excess (BE), lactate (Lac), haematocrit (Hct) and haemoglobin (Hb), with PaO_2_, PaCO_2_ and pH corrected to body temperature. The A-a gradient was calculated, as also reported by Gaudio et al. (2020b), from the formula (Sarkar, Niranjan & Banyal 2017):

$${\text{P(A-a)O}}_{\text{2}}{\text{=FIO}}_{\text{2}}{\text{(PB-PH}}_{\text{2}}{\text{O)-PaCO}}_{\text{2}}{\text{/RQ-PaO}}_{\text{2}}$$

where FIO_2_ is the fractional inspired oxygen (0.209), PB the barometric pressure (millimetre of mercury [mmHg]), PH_2_O the water vapour pressure of saturated air in the alveoli (mmHg) and RQ the respiratory quotient. Barometric pressure was measured by means of a calibrated portable barometer at the beginning of each immobilisation (Model CPG2300, Mensor Corporation, US). The PH_2_O in the alveoli and RQ were used at constant values of 47 mmHg (Christie & Loomis 1932) and 1 (standard RQ for healthy ruminants) (Kim et al. 2013), respectively.

At the end of monitoring, the overall quality of immobilisation was subjectively scored (immobilisation score) and the dart wound medically treated. The animal was transported back to its respective enclosure, positioned in sternal recumbency, and naltrexone was intravenously administered to antagonise etorphine’s effects. The time from naltrexone injection to the first sign of the animal becoming responsive (first sign of recovery) and the time until when the animal lifted its head (time to head up), stood up (time to standing) or started walking (time to walking) were measured and recorded. Recovery phase (from antagonist administration to the animal walking and being fully alert) was subjectively scored. Time and scores were based on those previously published by Gaudio et al. (2020b). All scores were assigned by the same blinded observer by means of the above-mentioned subjective scoring system (Table 1).

### Statistical analysis

Sample size calculation was performed by power analysis using the means and standard deviations of the paired differences of cardiorespiratory variables (i.e. PaO_2_, PaCO_2_ and A-a gradient) from previous research (Pfitzer et al. 2020) with an α = 0.05 and power = 0.8. For all data, median (range) (scores) or mean ± standard deviation (s.d.) (parametric data: clinical physiological variables, arterial blood gas variables, A-a gradients, induction and recovery times) was calculated. Distribution type was tested by using the Shapiro–Wilk test. Non-normally distributed data were log-transformed (lactate). Parametric data were analysed by using two-way analysis of variance (ANOVA) with fixed effects of time (physiological variables were measured at 5, 10, 15, 20, 25, 30, 35 and 40 min; arterial blood variables were measured at 5, 10, 15, 20 and 30 min) and treatment. As the same animals were administered each treatment, a repeated measures analysis was carried out. Bonferroni correction was used to perform *post hoc* pairwise comparisons (Bonferroni adjusted alpha level: 0.0018 and 0.01 for physiological and arterial blood variables, respectively). Subjective non-parametric data (induction, immobilisation and recovery score) were analysed by using Mann–Whitney and Kruskal–Wallis test. Data analysis was performed by using Statistical Analysis System (SAS) version 9.3 software. Values of *p* < 0.05 were deemed significant.

### Ethical considerations

The study was approved by the Wildlife Pharmaceuticals Animals Ethics Committee (Ethical clearance number: WPAEC-2018-AZAPBLES-25-B).

## Results

All animals were deemed as healthy on the basis of the veterinary health check and had an average weight of 56.9 ± 2.4 kg. Induction and recovery times evaluated in this study are reported in Table 2. No inter-treatment difference was found in mean time to first signs and induction time between HE and LE. Rapid immobilisation of all animals was achieved following the administration of either treatment (median induction score: 1, excellent). During maintenance, no inter-treatment difference in immobilisation score was found at any time point. The immobilisation plane was deemed as deep in both HE (median immobilisation score: 4) and LE (median immobilisation score: 3.5). Immobilisation score peaked at 20 min in HE (*p* = 0.025) and increased (*p* = 0.03) over time between 15 min and 20 min with LE. No inter-treatment difference was recorded for average first sign of recovery, time to head up, standing and walking. Time to standing and walking was always the same in both treatments. Recovery was scored as excellent in both treatments with blesbok showing minimal to no ataxia and getting up at first attempt. No post-immobilisation mortality or re-narcotisation was documented in any of the animals.

**TABLE 2:** Mean ± standard deviation of induction and recovery times and median score (range) of quality of induction, immobilisation and recovery of 12 female blesbok administered two etorphine–azaperone combinations characterised by different etorphine dose.†

Times and scores	HE	LE
Time to first sign (minutes)	1.8 ± 0.8	1.71 ± 0.45
Induction time (minutes)	3.2 ± 1.9	3.02 ± 0.43
First signs of recovery (minutes)	0.4 ± 0.1	0.35 ± 0.1
Time to head up (minutes)	0.5 ± 0.2	0.5 ± 0.2
Time to standing (minutes)	1.2 ± 0.3	1.1 ± 0.4
Time to walking (minutes)	1.2 ± 0.3	1.1 ± 0.4
Induction score	1 (1–2)	1 (1–2)
Immobilisation score	4 (3–4)	3.5 (2.5–4)
Recovery score	1 (1–1)	1 (1–1)

Source: Adapted from see the full reference list of the article Gaudio, E., Laubscher, L.L., Pfitzer, S., Raath, J.P., Hoffman, L.C. & Benedictis, G.M., 2020b, ‘Immobilisation quality and cardiopulmonary effects of etorphine alone compared with etorphine -azaperone in blesbok (Damaliscus pygargus phillipsi)’, *Veterinary Anaesthesia and Analgesia* 47(4), 528–536. https://doi.org/10.1016/j.vaa.2019.10.012, for more information

HE, high etorphine-azaperone; LE, low etorphine-azaperone.

† , HE (etorphine 0.09 mg kg^-1^, azaperone 0.35 mg kg^-1^) and LE (etorphine 0.05 mg kg^-1^ and azaperone 0.35 mg kg^-1^). At the end of the 40-min immobilisation period, naltrexone at a rate of 20 mg naltrexone per 1 mg etorphine was intravenously administered to antagonise etorphine.

Physiological variables and immobilisation scores measured over the 40-min immobilisation period after HE and LE administration are reported in Table 3. Overall mean values of PR and PE’CO_2_ were higher (*p* < 0.0001 and *p* < 0.028, respectively) with HE compared with LE. No intra-treatment differences from values at 5 min were noted in PR with HE, whereas in LE, PR decreased at 35 min (*p* = 0.02) and reached its lowest value at 40 min (*p* = 0.002). Intra-treatment differences were observed in PE’CO_2_ in HE at 20 min (*p* = 0.01) when values increased from the start of monitoring, whereas in LE, values increased at 20 min (*p* = 0.039) and peaked at 40 min (*p* = 0.019). Mean values of ƒ_R_ and SpO_2_ were higher (*p* = 0.006 and 0.005, respectively) after LE administration than those after HE administration. A decrease in ƒ_R_ from values recorded at 5 min was documented at 40 min (*p* = 0.034) with HE, whereas no intra-treatment differences were recorded with LE. No intra-treatment differences were found for SpO_2_ values in either treatment at any time point from starting values. Mean RT, SAP, MAP and DAP were not significantly different between HE and LE. Also, no intra-treatment differences from values at 5 min were recorded for the same variables with either HE or LE.

**TABLE 3:** Mean ± standard deviation of physiological variables and median score (range) of quality of immobilisation recorded at 5-min intervals during 40-min immobilisation of 12 female boma-acclimatised blesbok administered with two etorphine–azaperone combinations.†

Variable	Time points (minutes)																	
	5		10		15		20		25		30		35		40		Overall	
	HE	LE	HE	LE	HE	LE	HE	LE	HE	LE	HE	LE	HE	LE	HE	LE	HE	LE
PR (beats minute^-1^)	60 ± 19	60 ± 16	55 ± 14	47 ± 12	53 ± 14	44 ± 11	53 ± 14	43 ± 11	53 ± 15	42 ± 8	51 ± 14	41 ± 6	46 ± 12	39 ± 5*	45 ± 13	36 ± 4*	52 ± 15†	44 ± 11†
ƒ_R_ (breaths minute^-1^)	19 ± 4	21 ± 5	17 ± 3	18 ± 4	16 ± 5	16 ± 4	15 ± 5	17 ± 4	14 ± 3	16 ± 4	15 ± 3	17 ± 4	14 ± 5	16 ± 3	13 ± 4*	15 ± 4	15 ± 4**	17 ± 4**
SpO_2_ (%)	87 ± 8	93 ± 5	89 ± 5	94 ± 7	92 ± 7	92 ± 8	89 ± 9	92 ± 6.5	94 ± 7	94 ± 6	94 ± 6	94 ± 5	91 ± 5	96 ± 4	94 ± 6	96 ± 5	91 ± 7**	94 ± 6**
PE’CO_2_ (mmHg)	57 ± 6	55 ± 5	61 ± 5	57 ± 7	62 ± 4	60 ± 5	64 ± 5*	62 ± 6*	62 ± 6	62 ± 3	63 ± 4	61 ± 4	63 ± 4*	62 ± 4	62 ± 4	63 ± 6*	62 ± 5**	60 ± 6**
RT (°C)	39.0 ± 0.4	39.0 ± 0.5	39.0 ± 0.4	39.0 ± 0.5	38.9 ± 0.5	39.0 ± 0.6	38.8 ± 0.5	38.9 ± 0.5	38.8 ± 0.6	38.8 ± 0.5	38.8 ± 0.6	38.8 ± 0.5	38.7 ± 0.6	38.7 ± 0.5	38.6 ± 0.6	38.7 ± 0.5	38.8 ± 0.5	38.9 ± 0.5
SAP (mmHg)	136 ± 7	138 ± 11	133 ± 13	133 ± 10	128 ± 9	131 ± 11	126 ± 8	127 ± 10	123 ± 9	126 ± 12	125 ± 13	129 ± 11	127 ± 12	128 ± 10	126 ± 10	129 ± 10	128 ± 11	130 ± 11
DAP (mmHg)	103 ± 10	106 ± 7	102 ± 10	101 ± 8	98 ± 8	99 ± 10	95 ± 7	95 ± 9	93 ± 9	95 ± 9	94 ± 10	96 ± 7	95 ± 9	95 ± 9	95 ± 9	97 ± 10	97 ± 9	98 ± 9
MAP (mmHg)	119 ± 10	120 ± 8	114 ± 13	116 ± 8	113 ± 12	113 ± 10	108 ± 7	109 ± 9	106 ± 9	109 ± 10	107 ± 11	109 ± 9	109 ± 10	110 ± 10	109 ± 8	112 ± 9	111 ± 11	112 ± 9
Immobilisation score	3 (3–4)	3 (3–4)	4 (3–4)	4 (3–4)	4 (3–4)	4 (4–4)*	4 (3–4)*	4 (3–4)*	4 (3–4)	4 (3–4)	4 (3–4)	4 (3–4)	4 (3–4)	4 (3–4)	4 (3–4)	3 (3–4)	4 (3–4)	4 (3–4)

PR, pulse rate; ƒ_R,_ respiratory rate; SpO_2,_ peripheral oxygen-haemoglobin saturation; PE’CO_2,_ end-tidal carbon dioxide; RT, rectal temperature; SAP, systolic arterial blood pressure; DAP, diastolic arterial blood pressure; MAP, mean arterial blood pressure; HE, high etorphine; LE, low etorphine.

* , Indicates significant differences (*p* < 0.05) from values at 5 min;

** , indicates significant differences (*p* < 0.05) between treatments.

† , HE: 0.09 mg kg^-1^ etorphine and 0.35 mg kg^-1^ azaperone; LE: 0.05 mg kg^-1^ etorphine and 0.35 mg kg^-1^ azaperone.

Arterial blood values measured over the 40-min immobilisation are reported in Table 4. No overall differences were noted between HE and LE with regard to mean pH, PaO_2_, BE, Hct and Hb values. Mean PaCO_2_ and HCO_3_ were higher (*p* < 0.002 and 0.04, respectively) with HE than with LE. Mean lactate was higher with LE than with HE (*p* < 0.008). Also, pH, PaO_2_, PaCO_2_, HCO_3_ and BE showed no intra-treatment difference from initial values with either HE or LE. No difference from initial values were documented after HE in Hct and Hb, whereas they decreased at 30 min (*p* = 0.006 and 0.005, respectively) after LE administration.

**TABLE 4:** Mean ± standard deviation of blood gas variables recorded during immobilisation of 12 female boma-acclimatised blesbok administered with two etorphine–azaperone combinations.†

Variable	Time points (minutes)											
	5		10		15		20		30		Overall	
	HE	LE	HE	LE	HE	LE	HE	LE	HE	LE	HE	LE
PaO_2_ (mmHg)	43.0 ± 11.7	49.1 ± 13.0	40.0 ± 12.2	43.6 ± 10.3	39.7 ± 8.6	41.9 ± 8.4	41.4 ± 7.8	43.2 ± 6.9	47.6 ± 6.6	52.5 ± 9.8	42.2 ± 9.8	46.1 ± 10.4
PaCO_2_ (mmHg)	54.8 ± 3.8	51.6 ± 4.9	57.3 ± 4.2	53.8 ± 3.0	59.0 ± 4.3	55.7 ± 3.6	59.4 ± 3.7	57.6 ± 2.9	59.8 ± 4.9	56.2 ± 2.5	58.0 ± 4.5**	55.0 ± 3.9**
A-a gradient (mmHg)	39 ± 9	36 ± 11	40 ± 9	39 ± 10	38 ± 7	39 ± 10	36 ± 6	36 ± 9	29 ± 5	28 ± 11	36 ± 8	35 ± 11
HCO_3_ (mmol L^-1^)	33.2 ± 3.1	31.7 ± 1.5	33.4 ± 2.9	32.3 ± 2.4	33.7 ± 3.1	32.6 ± 2.5	33.9 ± 2.8	33.5 ± 1.4	33.9 ± 3.1	33.3 ± 1.8	33.6 ± 2.9**	32.7 ± 2.0**
BE (mmol L^-1^)	8.9 ±3.7	7.5 ± 1.9	8.7 ±3.3	7.9 ± 2.9	8.9 ±3.5	8.0 ± 2.7	9.1 ±3.1	8.8 ± 1.7	9.1 ±3.4	8.8 ± 2.0	8.9 ± 3.3	8.2 ± 2.3
pH	7.40 ± 0.04	7.41 ± 0.05	7.38 ± 0.04	7.39 ± 0.03	7.37 ± 0.04	7.38 ± 0.03	7.37 ± 0.03	7.38 ± 0.03	7.37 ± 0.04	7.39 ± 0.02	7.38 ± 0.04	7.39 ± 0.03
Lac (mmol L^-1^)	1.3 ± 0.9	2.0 ± 2.3	1.1 ± 0.6	1.75 ± 1.9	1.0 ± 0.5	1.6 ± 1.7	0.9 ± 0.4	1.5 ± 1.6	0.8 ± 0.4	1.3 ± 1.4	1.0 ± 0.6**	1.6 ± 1.7**
Hct (%)	29 ± 3	29 ± 3	28 ± 3	27 ± 3	27 ± 2	26 ± 2	26 ± 3	26 ± 2	26 ± 3	25 ± 2*	27 ± 3	27 ± 3
Hb (mmol L^-1^)	6.2 ± 0.6	6.2 ± 0.6	5.9 ± 0.6	5.6 ± 0.6	5.7 ± 0.5	5.5 ± 0.5	5.5 ± 0.5	5.4 ± 0.5	5.5 ± 0.6	5.3 ± 0.5*	5.7 ± 0.6	5.6 ± 0.6

PaO_2_, partial pressure of oxygen; PaCO_2_, partial pressure of carbon dioxide; HCO_3_^-^, bicarbonate; BE, base excess; Lac, lactate; Hct, haematocrit; Hb, haemoglobin; HE, high etorphine; LE, low etorphine.

* , Indicates significant differences (*p* < 0.05) from values at 5 min;

** ,indicates significant differences (*p* < 0.05) between treatments.

† , HE: 0.09 mg kg^-1^ etorphine and 0.35 mg kg^-1^ azaperone; LE: 0.05 mg kg^-1^ etorphine and 0.35 mg kg^-1^ azaperone.

No difference was recorded in mean A-a gradient values between HE and LE. Furthermore, A-a gradient did not change from starting values in both treatments.

## Discussion

Both drug combinations provided a complete immobilisation in all the blesbok for the entire duration of monitoring. Compared with other ungulate immobilisation protocols, induction time (3.2 ± 1.9 and 3.0 ± 0.4 min with HE and LE, respectively) was quick and uneventful. Induction time is considered one of the most important factors when immobilising wild animals. Keeping induction time as short as possible is desirable when capturing antelope because this is believed to translate into a reduction in capture-related morbidity (Meyer et al. 2008a; Meyer et al. 2008b). Under field conditions, a short induction time also reduces the risks of animals being attacked by predators and other herd members (Pfitzer et al. 2020). In this study, the reduced etorphine dose did not prolong the induction time. This seems to point out that the etorphine dose used in LE could be sufficient to provide a quick induction in this antelope species.

It has been reported that opioids, either used as a sole agent or in combination with other drugs, can lead to hypertension in wild herbivores (Buss et al. 2016; Gaudio et al. 2020b; Hattingh et al. 1994; Heard et al. 1996; Pfitzer et al. 2020). The mechanisms behind this are not yet completely clear, but one hypothesis is that hypertension might be induced by a sympathetic nervous system (SNS) reflex response to the drug-induced hypoxia and hypercapnia. It could also be hypothesised that hypertension is caused by a direct activation of receptors in the SNS by opioids (Daniel & Ling 1972; Fuerstein & Sirén 1987; McQueen 1983). In an attempt to reduce hypertension, etorphine is often combined with azaperone because the latter is reported to have vasodilating properties (Kock & Burroughs 2012; Riviere & Papich 2009). Hypertension has been defined as a MAP > 95 mmHg in anaesthetised goats, a domestic ruminant of comparable size to that of blesbok (Thatcher & Keith 1986). In the present study, both HE and LE produced a similar degree of hypertension (MAP: 110.5 ± 10.5 mmHg and 111.9 ± 9.3 mmHg in HE and LE, respectively). This finding indicates that the decrease in etorphine dose was not enough to avoid the development of hypertension. Also, azaperone’s α_1_-adrenergic antagonistic effect, at the dose used, might not have been enough to counteract etorphine- and SNS-related side effects. Whether normotension during immobilisation could be achieved by using a higher azaperone dose remains to be tested. Also, it has to be considered that an increase in azaperone dose might lead to the manifestation or worsening of some of its side effects, such as excessive sedation, seizures and catalepsy (The European Agency for the Evaluation of Medicinal Products 1998). A recent study in conscious dogs highlighted how acepromazine, another dopaminergic drug, leads to a decrease in blood pressure through cardiac output impairment rather than a drop in systemic vascular resistance (Rangel et al. 2020). Azaperone’s effects on cardiac output and systemic vascular resistance in antelopes are not known yet, and therefore further research is necessary to clarify azaperone’s possible dose-dependent adverse and opioid-sparing effects.

Blesbok appeared to be bradycardic (mean PR in awake resting blesbok: 104 beats min^-1^; Du Plessis 2018) after the administration of each drug combination (52.1 ± 14.5 4 beats min^-1^ and 43.7 ± 11.4 beats min^-1^, with HE and LE, respectively). Bradycardia is believed to be caused by the baroreceptor-mediated reflex secondary to the increase in arterial blood pressure as a result of etorphine (Alexander & De Cuir 1962). Another possible explanation could be sought in the medullary vagal stimulation induced by opioids (Bowdle 1998). Furthermore, it is known that opioids can directly interact with opiate receptors, both in the nervous system and in the myocardium, leading to cardio-depressant effects (Gautret & Schmitt 1984; Roquebert & Delgoulet 1988). In the present study, bradycardia was more profound when LE was administered (43.7 ± 11.4 and 52.1 ± 14.5, with LE and HE, respectively). Considering that MAP was similar between treatments, the difference in blood pressure is not deemed to be the primary cause for inter-treatment difference in PR. Unfortunately, the reason why animals were more bradycardic with LE remains poorly understood.

During chemical immobilisation, etorphine-induced activation of µ-opioid receptors in the respiratory centres causes hypoventilation (Kock & Burroughs 2012). In addition, the activation of the same receptors in the brainstem, and on the aortic arch and carotid bodies, can cause a desensitisation of these to hypercapnia, hypoxaemia and acidaemia (Buss & Meltzer 2001; McCrimmon & Alheid 2003). Hypoventilation is characterised by an increase in PaCO_2_ with a decrease in PaO_2_, both of which were recorded in the present study. In fact, mean PaCO_2_ was suggestive of hypercapnia (defined as PaCO_2_ > 45 mmHg; Bautista & Akca 2013) with both HE and LE. Although hypercapnia was significantly greater with HE than with LE (mean PaCO_2_: 58.0 ± 4.5 mmHg and 55.0 ± 3.9 mmHg), its inter-treatment difference was not deemed as clinically relevant (only 3 mmHg overall difference). The greater hypercapnia seen with HE could be associated with the lower ƒ_R_ than what was recorded with LE (15 ± 4 breaths minute^-1^ and 17.1 ± 4.3 breaths minute^-1^). Although there was an absence of inter-treatment difference between mean PaO_2_ values (42.2 ± 9.8 mmHg and 46.1 ± 10.4 mmHg in HE and LE, respectively), both treatments led to marked hypoxia (PaO_2_ < 60 mmHg; Grimm et al. 2015). These results indicate that a reduction of the etorphine dose in this drug combination does not equate to a clinically relevant improvement in ventilation. Also, spirometry could not be carried out in the present study, thus limiting interpretation of results.

Prolonged hypoventilation may lead to atelectasis of some parts of the lungs, thus impairing gas exchange and widening the A-a gradient (Hedenstierna & Edmark 2010; Sarkar et al. 2017). The A-a gradient is a measure of the difference in oxygen concentration between alveoli and arterial blood (McFarlane & Imperiale 1994). Large differences between these values (or a large A-a gradient) are predictive of a clinically relevant defect in oxygen diffusion from the alveoli into the blood (Bateman 2008) as a consequence of the compromised alveolocapillary unit (Sarkar et al. 2017). Furthermore, Meyer et al. (2015) state that elevated A-a gradients, with normal to mildly elevated PaCO_2_, indicate that hypoxaemia may be the result of pulmonary hypertension because of vasoconstriction, formation of oedema and ventilation perfusion (V̇/Q̇) mismatch rather than hypoventilation alone in goats administered etorphine. Similar effects were also reported in etorphine-immobilised sheep and goats in a study by Izwan et al. (2018). In ruminants, an A-a gradient higher than 10 mmHg indicates suboptimal alveolar–arteriolar oxygen exchange (Neary, Garry & Raabis 2014). In the present study, both treatments showed mean A-a gradients greater than 10 mmHg (HE and LE: 36.1 ± 8.1 mmHg and 35.3 ± 10.6 mmHg, respectively) indicating alveolar-capillary unit gas diffusion impairment. The A-a gradient widening recorded in the present study could be because of the direct effect of etorphine at the level of the pulmonary capillaries. As no inter-treatment difference was found with regard to mean A-a gradient, it can be deduced that LE did not prevent or reduce gas-exchange impairment. At present, no information is available regarding possible effects of azaperone on alveolar–arteriolar gas exchange; therefore, whether its presence might have played a role is not known.

With both treatments, the plane of immobilisation was deemed adequate in all animals, as it was deep enough to induce loss of evaluable reflexes (e.g. panniculus reflex, palpebral reflex) with good muscle relaxation. Handling of the animals was deemed as safe for the research team for the entire duration of monitoring. Immobilisation depth peaked between 15 min and 20 min post-recumbency with both HE and LE. This finding may be because of azaperone taking full effect within 10–20 min after IM administration (Lees & Serrano 1976). The lower etorphine dose tested in the present study in combination with azaperone provided an equivalent immobilisation quality compared with the higher etorphine dose combination, disproving the hypothesis that a lower dose of etorphine would result in a poorer quality of immobilisation.

Excitement and stress during darting and the induction phase may lead to an animal developing acidaemia, high lactate levels and hyperthermia (Andrade et al. 2019; Meyer et al. 2008a; Meyer et al. 2008b; Sharkey & Wellman 2015). In the present study, none of the treatments led to any of these abnormalities. Blood pH and rectal temperature were comparable between treatments, but lactate levels were higher with LE compared with HE (1.6 ± 1.7 mmol L^-1^ vs. 1.0 ± 0.6 mmol L^-1^). This is an interesting finding as immobilisation time and quality were not different between treatments. Although higher, these lactate values were within normal limits (< 2 mmol L^-1^) (Pang & Boysen 2007), and deemed as not clinically relevant.

A quick recovery phase is crucial when immobilising wildlife in the field, as the animal needs to be fully awake and aware of its surrounding in the shortest time possible to avoid predation and possible environmental dangers. A common undesired complication is prolonged and fractious recoveries, which sometimes are common in antelope. In the present study, recovery was always rapid and uneventful after naltrexone administration with both treatments (time to standing: 1.2 ± 0.3 and 1.1 ± 0.4 min, with HE and LE, respectively). This finding indicates that the etorphine doses, as used in this study, did not affect recovery time.

There are some limitations to this study that should be addressed in future research. The lack of endotracheal intubation could have allowed for gas coming from the rumen to influence PE’CO_2_ values recorded in the nasopharynx (Ding et al. 2010), and it is recommended that animals be intubated in future studies. In the present study, animals were not intubated because of the fact that they maintained laryngeal reflexes during immobilisation. Furthermore, measurement of V^˙^/Q^˙^ ratios, pulmonary vascular pressures, cardiac output and tidal volume was not feasible in this study, and therefore interpretation of the effects of these combinations is not as comprehensive as could be achieved. Finally, it has to be mentioned that in the present study, animals were darted within bomas, and induction times, physiological changes and quality of immobilisation might differ when using these combinations in free-ranging blesbok.

In conclusion, both treatments resulted in a quick immobilisation and an uneventful recovery of all animals. Immobilisation quality was good and allowed for easy handling of all the animals with both HE and LE. The main hypothesis of this study was disproved because the lower etorphine dose did not provide a sufficient improvement in cardiorespiratory variables compared with the high etorphine dose combination. Hypertension, bradycardia, hypercapnia and hypoxaemia following the administration of both drug combinations were still evident. Also, reducing the etorphine dose did not worsen the quality of immobilisation. These findings highlight that further studies are needed to better understand the physiological effects of the many different drug doses and combinations that are used in wildlife immobilisation.
